# Identifying Family Caregivers in Hospitals: Overcoming Challenges

**DOI:** 10.1093/geroni/igaf122.039

**Published:** 2025-12-31

**Authors:** Alycia Bristol

**Affiliations:** University of Utah, Salt Lake City, Utah, United States

## Abstract

During hospitalization, family caregivers (family, friends, or other support persons) play a vital role in supporting positive health outcomes for patients. Family caregivers often feel unprepared and unsure about providing care after hospital discharge. To support identification of caregivers’ needs at discharge, we are adapting and testing the Family Readiness for Hospital Discharge Scale (FamRHDS). Testing of the FamRHDS scale centers on recruitment of patients and caregivers at hospital discharge. During our study, we have observed changes in recruiting caregivers post-COVID. While we successfully recruited patients and caregivers via telephone and text during COVID, we now face challenges due to non-functional or absent telephones in patient rooms and a fast-paced discharge process that often limits the time and availability of caregivers. Moreover, identification of caregivers within the electronic health record remains a challenge. This has led to decreased response rates for caregiver recruitment, contrasting with higher patient recruitment rates. To address this, we have shifted to maintaining an in-person presence within the hospital. We meet with patients to identify a preferred primary caregiver. When engaging with caregivers, we prioritize communicating our intention to understand their needs, emphasizing the practical impact and applicability of our work, rather than focusing on research for its own sake. Furthermore, we have established relationships with clinicians and administrators to support the identification of potential participants and raise awareness of our study. From our work, we have learned the importance of focusing on the utility of our research plan to better align with caregivers’ needs.

